# Prevention of Hepatitis B Recurrence in Liver Transplant Patients Using Oral Antiviral Therapy without Long-Term Hepatitis B Immunoglobulin

**DOI:** 10.5812/kowsar.1735143X.717

**Published:** 2011-08-01

**Authors:** Joseph Ahn, Stanley Martin Cohen

**Affiliations:** 1 Department of Hepatology, Loyola University Medical Center, Maywood, USA

**Keywords:** Hepatitis B, Liver transplantation, Immunoglobulin

## Abstract

**Background:**

Small studies have suggested that nucleos(t)ide analogue therapy (NAT) with reduced hepatitis B immunoglobulin (HBIG) duration may be efficacious in preventing post-liver transplantation (LT) HBV recurrence.

**Objectives:**

This larger study evaluates the use of NAT with short term (< 6 mo) or no HBIG for prevention of post-LT HBV recurrence.

**Patients and Methods:**

All HBV patients undergoing LT at a university transplant center between 2002 and 2007 were identified retrospectively. Patient demographics, medication regimen, and adverse events were noted. The primary endpoint was HBV recurrence and secondary endpoints were graft and patient survival.

**Results:**

28 study patients were identified. Of these 28 patients, 4 (14%) received no HBIG, 6 (22%) received only inpatient HBIG, and 18 (64%) received inpatient HBIG and outpatient HBIG. 16 of the 28 patients (57%) received combination NAT and 12 patients (43%) received single NAT. At a median time of 15.5 months (range 9-24 months) post-LT, 4 of the 28 patients (14%) had recurrent HBV. Of those patients with recurrent HBV, 3 received both inpatient and outpatient HBIG and 1 received no HBIG. All cases of HBV recurrence were associated with noncompliance.

**Conclusions:**

NAT with short-term or no HBIG was efficacious and safe in preventing post-LT HBV. All compliant patients were HBV-free, including 9 patients who received no HBIG or only inpatient HBIG. Additional studies using NAT without HBIG appear justified.

## 1. Background

The number of patients with hepatitis B (HBV) listed for liver transplantation (LT) in the United States has decreased by 37% since 2000, presumably due to the availability of effective oral antiviral therapy [1]. However, the more than 350 million people infected worldwide continue to make HBV and associated hepatocellular carcinoma important indications for liver transplantation. Therefore, prevention of HBV recurrence in patients undergoing LT will remain an important challenge worldwide. Historically, many transplant centers have been reluctant to offer LT for HBV patients due to the high risk of recurrence leading to graft failure, increased patient mortality, and the need for re-transplantation. After the protective ability of long-term, high-dose hepatitis B immunoglobulin (HBIG) was recognized, however, HBV became an accepted indication for LT [2]. HBIG is expensive and carries an HBV recurrence risk of 15-25% [3]. Several small studies have explored the effects of alterations to the administration route, dose, total amount, frequency, and duration of HBIG treatment while incorporating the use of oral antiviral agents with nucleos(t)ide analogue therapy (NAT) [4][5][6][7]. The transition from HBIG to NAT would provide a significant reduction in the costs, side effects, and inconveniences that are associated with HBIG, without sacrificing efficacy.

## 2. Objectives

The purpose of this study was to examine the efficacy of NAT with minimal or no HBIG in preventing HBV recurrence after LT in a relatively large number of patients.

## 3. Patients and Methods

We reviewed the medical records of all patients with hepatitis B who underwent liver transplantation between 2002 and 2007 at Rush University Medical Center in Chicago, Illinois. Hepatitis B diagnosis was defined by a positive HBV surface antigen (HBsAg) and HBV DNA. HBV patients undergoing liver transplantation for fulminant hepatic failure, hepatocellular carcinoma, and end stage liver disease were included. Patients co-infected with hepatitis C (HCV), hepatitis D (HDV), or human immunodeficiency virus (HIV) were also included in our analysis. Patients who suffered peri-operative death and did not receive HBIG or antiviral therapy were excluded from analysis.

The following data were obtained for each patient:

1) Clinical information- gender, age, ethnicity/race; date, indication for transplantation, Model for End stage Liver Disease (MELD) score at transplantation; follow-up time, time to HBV recurrence, graft and patient survival, re-transplantation, and cause of death.

2) Virological and serological data- pre-LT HBsAg, HBV e antigen, HBV e antibody, HBV DNA, HCV antibody, HCV RNA, HDV antibody, HIV antibody, liver function tests; post-LT HBsAg and HBV DNA, liver function tests.

3) Treatment- pre-LT antiviral NAT regimen, post-LT NAT regimen, HBIG prophylaxis regimen, post-LT immunosuppression, adverse reactions and compliance to NAT. Resistance testing for mutations, including the YMDD mutation, was not universally obtained.

The primary endpoint of this study was HBV recurrence, as defined by a positive test for HBV DNA in the blood. The secondary endpoints included the safety of NAT and the survival of grafts and patients.

### 3.1. Anti HBV Therapy

We noted the pre-transplant antiviral treatment regimen for each patient, including drug, dose, frequency, and duration of treatment. Patients were divided into 3 groups based on HBIG utilization. Group 1 received no HBIG. Group 2 received only inpatient HBIG daily for up to 7 days. Group 3 received inpatient HBIG daily for up to 7 days and outpatient HBIG monthly for up to 6 months. In all cases, the HBIG dose was 10,000 IU given intravenously. All patients received post-LT NAT of lamivudine, adefovir, or tenofovir, either singly or in combination. The differences in anti-HBV therapy reflected the evolving use of HBIG and NAT by the transplant team between 2002 and 2007. In general, more HBIG was used in 2002 and less or no HBIG was used in 2007.

### 3.2. Immunosuppressive Therapy

Patients were maintained on a standard immunosuppressive regimen with calcineurin inhibitor-based therapy (tacrolimus or cyclosporine). The doses of calcineurin inhibitors were adjusted based on tacrolimus and cyclosporine levels. Corticosteroids were used for immunosuppression induction and typically were tapered off by 6 months post liver transplantation. Any acute cellular rejection was confirmed by liver biopsy and treated with corticosteroid boluses.

### 3.3. Clinical Follow-up

Standard hematological, biochemical, and HBV serological and virological testing was performed regularly after transplantation. After 2006, HBV DNA testing was performed using Versant HBV 3.0 bDNA Assays (Bayer Health Care LLC, Tarrytown, NY). Prior to 2006, HBV DNA testing was performed using Roche HBV DNA Quantitation Kits (Roche Molecular Systems, Branchburg, NJ). Testing for the presence of HBV mutants was not routinely performed. Liver biopsy was obtained as clinically indicated for investigation of elevated liver function tests or for evaluation of graft dysfunction. Histological evaluation included immunohistochemical staining for HBsAg and HBV core antigen. Compliance was assessed by whether patients returned for clinical follow-up visits, refilled NAT prescriptions, and reported the use of NAT during clinic visits, as recorded in the medication lists of their clinical records.

### 3.4. Statistical Analysis

All data entry and collection was performed using Microsoft Excel 2003 (Microsoft). Statistical analysis was performed using SAS 8.2 (SAS Institute Inc.). We used Chi-square tests and Wilcoxon tests to compare patient groups. Approval from the institutional review board at Rush University Medical Center was obtained for the study.

## 4. Results

### 4.1. Patient Characteristics

32 of the 525 (6.1%) liver transplants performed between 2002 and 2007 at Rush University Medical Center in Chicago, Illinois were for HBV. Four patients who died peri-operatively and did not receive HBIG were excluded from analysis. Baseline characteristics are shown in Table 1. Two patients in group 2 had undetectable HBsAg but positive HBV DNA pre- LT. Eighteen of the patients included in the study (64%) were men, and 13 (47%) were white. Patients had a median age of 54.5 years. Of the 28 patients, 26 had cirrhosis, 9 had concomitant hepatocellular carcinoma, and 2 presented with fulminant hepatic failure. Group 1 comprised 4 patients who received no HBIG. Group 2 included 6 patients who received only daily inpatient HBIG. Group 3 comprised 18 patients who received daily inpatient HBIG for up to 7 days and monthly outpatient HBIG for up to 6 months.

**Table 1:** Baseline Characteristics

	**Group 1, No HBIG (n = 4)**	**Group 2, Inpatient HBIG (n = 6) **	**Group 3, Inpatient and Outpatient HBIG (n = 18) **	**P value**	**Total (n = 28)**
Gender, M a/F a	2/2	3/3	13/5	18/10	0.447
Age, y, median (range)	57 (43-73)	62 (47-71)	53 (39-72)	54.5 (39-73)	0.209
Ethnicity, No. (%)					0.459
Asians	0 (0)	1 (16)	5 (28)	6 (21)	
>African-Americans	0 (0)	2 (33)	3 (16)	5 (18)	
Hispanic	2 (50)	0 (0)	2 (11)	4 (14)	
White	2 (50)	3 (50)	8 (44)	13 (46)	
Indication for LT, No. (%)					
Cirrhosis	4 (100)	6 (100)	16 (89)	26 (93)	0.405
Liver Cancer	2 (50)	2 (33)	5 (28)	9 (32)	0.839
Fulminant hepatic failure	0 (0)	0 (0)	2 (11)	2 (7)	1
MELD a, mean score	19	22	22	21.9	0.93
sAg, No. (%)	4 (100)	4 (67)	18 (100)	26 (93)	
eAg, No. (%)	2 (50)	2 (33)	8 (44)	12 (43)	
eAb, No. (%)	1 (25)	4 (67)	7 (39)	12 (43)	
HBV DNA (+) at time of NAT^a^ initiation, No. (%)	1 (25)	3 (50)	14 (78)	18 (64)	
DNA level, IU/L, median	2230	179 (range 179 to 1 × 107)	25,375 (range 20 to 1 × 107)	23200 (range 20 to 1 × 107)	0.743
HCV Ab (+), No. (%)	2 (50)	1 (16)	1 (6)	4 (14)	0.063
HDV Ab (+), No. (%)	0 (0)	0 (0)	3 (17)	3 (11)	
HIV Ab (+), No. (%)	1 (25)	0 (0)	0 (0)	1 (4)	0.115

^a^ Abbreviations: F, female; LT, liver transplantation; M, male; MELD, model for end stage liver disease; NAT, nucleos(t)ide analogue therapy

### 4.2. HBV Prophylaxis 

18 patients (64%) received antiviral treatment with NAT prior to LT. Table 2 outlines the treatment characteristics. Prior to LT, 1 of the 4 patients in Group 1 had been on antiviral treatment for 2 months, 4 of the 6 patients in Group 2 had been on antiviral treatment for a median of 8.7 months (range 3-26.3 months), and 13 of the 18 patients in Group 3 had been on antiviral treatment for a median of 4.1 months (range 0.2-16.8 months). Pre-LT treatment for 13 of the 28 patients (46%) included monotherapy with lamivudine (12/13) or entecavir (1/13) for a median of 2.7 months (range 0.2-26.3 months), while 5 patients (18%) received a combination of antiviral medications (4 on lamivudine 100 to 150 mg and adefovir 10 mg daily, 1 on entecavir 0.5 mg and adefovir 10 mg daily) for a median of 12.7 months (range 4.3-16.8) before LT. All HBIG doses were 10,000 IU given intravenously. Group 1 received no HBIG. Group 2 received daily inpatient doses of HBIG for an average of 4 days and no outpatient monthly HBIG. Group 3 received daily inpatient doses of HBIG for an average of 7 days and monthly outpatient doses of HBIG for an average of 6 months. After LT, 3 patients in Group 1 received monotherapy with entecavir, lamivudine or adefovir and 1 patient in Group 1 received a combination of lamivudine and adefovir. In Group 2, 5 patients were treated with a combination of lamivudine and adefovir and 1 patient was treated with lamivudine alone. In Group 3, 9 patients received combination NAT consisting of lamivudine and adefovir, 1 patient received a combination of emcitritabine and tenofovir, and 8 patients received lamivudine monotherapy.

**Table 2:** Treatment Characteristics

	**Group 1, No HBIG (n = 4) **	**Group 2, Inpatient HBIG (n = 6) **	**Group 3, Inpatient and outpatient HBIG (n = 18) **	**Total (n = 28)**
Pre-LT a antiviral therapy, No. (%)	1 (25)	4 (67)	13 (72)	18 (64)
Monotherapy, No. (%)	1 (25)	3 (50)	9 (50)	13 (46)
Lamivudine, No. (%)	1 (25)	1 (16)	9 (50)	11 (39)
Entecavir, No. (%)	0 (0)	2 (33)	0 (0)	2 (7)
Combination therapy, No. (%)	0 (0)	1 (16)	4 (22)	5 (18)
Lamivudine + Adefovir, No. (%)	0 (0)	0 (0)	4 (22)	4 (14)
Entecavir + Adefovir, No. (%)	0 (0)	1 (16)	0 (0)	1 (4)
Pre-LT Rx b duration, median mo. (range)	2	8.7 (3–26.3)	4.1 (0.2–16.8)	4.2 (0.2–26.3)
HBIG a, 10,000 IU/IV dose				
Inpatient, daily, mean No. doses	0	4	7	5.1
Outpatient, monthly, mean No. doses	0	0	6	3.5
Post-LT Antiviral Therapy				
Monotherapy, No. (%)	3 (75)	1 (16)	8 (44)	12 (43)
Lamivudine	1 (25)	1 (16)	8 (44)	10 (36)
Adefovir	1 (25)	0 (0)	0 (0)	1 (4)
Tenofovir	1 (25)	0 (0)	0 (0)	1 (4)
Combination therapy, No. (%)	1 (25)	5 (83)	10 (56)	16 (57)
Lamivudine + Adefovir	1 (25)	5 (83)	9 (50)	15 (54)
Emcitritabine + Tenofovir	0 (0)	0 (0)	1 (6)	1 (4)

^a^ Abbreviations: HBIG, hepatitis B immunoglobulin; LT, liver transplantation

^b^ Rx, treatment

### 4.3. HBV Recurrence

Four of the 28 patients included in the study (14%) had HBV recurrence with positive HBsAg and a median HBV DNA of 9.4 × 106 IU/ml. Median time to HBV recurrence was 15.5 months (range 9.9-24.3 months). Table 3 outlines the risk factors for HBV recurrence.

**Table 3:** Risk Factors for Recurrence

	**Recurrence (n = 4)**	**Non-recurrence (n = 24)**	**Total (n = 28)**	**P value**
Pre-LT antiviral therapy, No. (%)	2 (50)	16 (67)	18 (64)	1
Combination, No. (%)	0 (0)	5 (21)	5 (18)	1
Monotherapy, No. (%)	2 (50)	11 (46)	13 (46)	1
Duration of Rx a, median mo.	2	2	2	0.341
Pre-LT DNA level, IU/L	1.7 × 10^6^ (range 7.1 × 10 ^5^ to 2.5 × 10 ^ 6^)	8113 (range 20 to ^1^ × 10 ^7^)	23200 (range 20 to^ 1^ × 10 ^7^)	0.213
HBIG Group				0.581
Group 1, No. (%)	1 (25)	3 (12)	4 (14)	
Group 2, No. (%)	0 (0)	6 (25)	6 (22)	
Group 3, No. (%)	3 (75)	15 (63)	18 (64)	
Noncompliance, No. (%)	4 (100)	0 (0)	4 (14)	< 0.0001
Post-LT Antiviral Therapy				
Monotherapy, No. (%)	1 (25)	11 (46)	12 (43)	0.1667
Lamivudine	0 (0)	10 (42)	10 (36)	
Adefovir	1 (25)	0 (0)	1 (4)	
Tenofovir	0 (0)	1 (4)	1 (4)	
Combination therapy, No. (%)	3 (75)	13 (54)	16 (57)	0.1875
Lamivudine + Adefovir	2 (50)	13 (54)	15 (54)	
Emcitritabine + Tenofovir	1 (25)	0 (0)	1 (4)	

^a^ Rx, treatment

The median pre-LT HBV DNA for patients with HBV recurrence was 1.7 × 106 IU/ml (range 7.1 × 105 to 2.5 × 106 IU/ml) versus 8113 IU/ml (range 20 to 1 × 107 IU/ml) for patients without HBV recurrence (P = 0.21). After LT, 3 of the 4 patients with HBV recurrence had been prescribed a combination of lamivudine and adefovir, and 1 patient was prescribed adefovir monotherapy. HBV mutation testing for NAT resistance was not available for these patients. All four patients with HBV recurrence had been noncompliant with prescribed antiviral treatment and follow-up. One patient did not return for any follow-up visits for 10 months. During that time, she did not take her NAT. A second patient was lost to follow-up for 9 months during which time he did not take his NAT. A third patient admitted to not taking her NAT when she was diagnosed with HBV recurrence. The fourth patient indicated that she did not take her NAT due to financial difficulties and was lost to follow-up for 21 months. When she returned for follow-up care, she was diagnosed with HBV recurrence.

**Figure 1:**
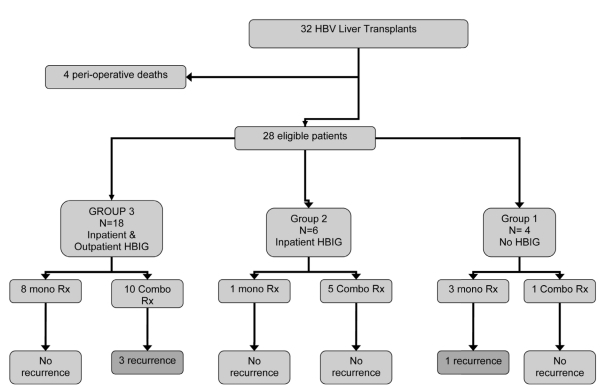
HBV Liver Transplant Patient Outcomes

24 of the 28 patients studied (86%) did not have recurrence, with negative HBV DNA at a median of 35.2 months post-LT (range 3.4-58 months). All 24 were compliant with the prescribed antiviral therapy and clinical follow-up. 13 of the 24 patients without HBV recurrence (54%) received combination NAT, with 12 receiving lamivudine and adefovir and 1 receiving emcitritabine and tenofovir. The remainder (11/24, 46%) received NAT monotherapy, with 10 receiving lamivudine and 1 receiving tenofovir. In Group 1, 1 of the 4 patients (25%) experienced HBV recurrence. In Group 2, no patients experienced HBV recurrence and in Group 3, 3 of the 18 patients (17%) experienced HBV recurrence. There were no statistical differences in recurrence with respect to HBIG prophylaxis (P = 0.15) There were also no differences in recurrence with respect to post-LT combination NAT (P = 0.19), post-LT NAT monotherapy (P = 0.17), or to pre-LT DNA levels (P = 0.21). Recurrence occurred only in patients who did not comply with the prescribed HBV prophylaxis regimen (P < 0.0001). The time between LT and recurrence was longer in Group 3 than in Group 1 (P = 0.029). All cases of HBV recurrence were effectively treated with combination NAT, all 4 patients with HBV recurrence were alive at follow-up a median 42.2 months after LT, and none of these patients experienced graft loss. Figure 1 provides an outline of patient outcomes.

### 4.4. Survival

Outcomes for patients in each HBIG group are shown in Table 4. Patients were followed for a median of 35.2 months (range 3.4-57.5 months). There were no differences between the 3 HBIG groups in time followed (P = 0.058), graft loss (P = 0.284), or death (P = 0.143). A total of 3 patients (14%) suffered graft loss unrelated to HBV recurrence; 1 due to primary nonfunction and 2 due to biliary cast syndrome. These 3 patients underwent repeat LT at a median of 99 days after the initial LT (range 19-366 days). There was 1 death unrelated to HBV recurrence that was due to cerebrovascular accident 306 days after LT.

**Table 4:** Outcome

	**Group 1, No HBIG a(n = 4) **	**Group 2, Inpatient HBIG (n = 6) **	**Group 3, Inpatient and outpatient HBIG (n = 18) **	**Total (n = 28)**	**P value**
Recurrence, No. (%)	1 (25)	0 (0)	3 (17)	4 (14)	0.581
Recur., median mo. (range)	9.9	NA	21 (8.8–24.4)	15.5 (8.8–24.4)	0.029
Follow up, median mo. (range)	16.8 (3.4–47.9)	23 (13.8–57.2)	42.9 (19.3–57.5)	35.2 (3.4–57.5)	0.0579
Death, No. (%)	1 (25)	0 (0)	0 (0)	1 (4)	0.143
Graft loss and repeat Transplantation, No.(%)	1 (25)	1 (16)	1 (6)	3 (14)	0.284

^a^ Abbreviations: HBIG, hepatitis B immunoglobulin

### 4.5. Adverse Events and Safety

There were no reports of significant adverse events associated with HBIG use. There were no reports of significant adverse events associated with NAT. There was no new diagnosis of renal insufficiency or any worsening underlying renal insufficiency associated with antiviral treatment. Ten of the 16 patients (63%) on combination NAT had underlying chronic insufficiency with resultant renal-adjusted dosing.

## 5. Discussion

Although the mechanism of HBIG in preventing HBV recurrence post-LT is not fully understood, its ability to prevent recurrence in 75% to 85% of patients transformed HBV from a contraindication to a readily accepted indication for LT worldwide [2][3][8]. However, the use of HBIG has been limited due to its indefinite use, significant cost, parenteral administration, persistent risk of recurrence, and development of HBV surface antigen (HBsAg) escape mutations [3][9][10][11]. Adverse effects include headaches, myalgias, flushing, pain, and even reports of mercury poisoning [12]. These limitations of HBIG have pushed investigators to explore lower cost and more effective alternatives to prevent post-LT HBV recurrence. These have included adjusting HBIG doses based on the HBV surface antibody titer, intramuscular administration of HBIG, and HBIG treatment of finite duration [4][5][8]. Oral antiviral therapy with NAT offered an alternative to the costs and side effects associated with HBIG when lamivudine became available in 1995. However, the elevated risk of HBV recurrence with lamivudine monotherapy became apparent when initial reports of less than 10% HBV recurrence at 1 year were followed by reports of 45% recurrence at 3 years, due to the development of the YMDD mutation [13][14].

The next phase in HBV recurrence prophylaxis was the combination of lamivudine with HBIG. Markowitz et al. reported that 14 patients who received lamivudine and high-dose IV HBIG had no recurrence at 13.2 months [15]. Yao et al. were able to convert 10 patients on lamivudine from high-dose IV HBIG to IM HBIG, with only 1 HBV recurrence due to noncompliance 15.6 months after the change was made [16]. Other researchers using combination HBIG and lamivudine reported a lower than 10% risk of HBV recurrence more than 3 years after LT [17][18]. Several small studies have evaluated the use of newer NAT with and without HBIG in preventing post-LT HBV recurrence. Nath et al. reported that 14 patients who received 7 days of high-dose IV HBIG with tenofovir or lamivudine and adefovir experienced no clinically significant HBV recurrence [7]. Schiff et al. more recently reported that 23 patients who received lamivudine and adefovir before and after LT without HBIG experienced no HBV recurrence during 9 months of follow-up [19]. Neff et al. reported that 10 patients on combination lamivudine and adefovir had no HBV recurrence at a mean of 31 months after discontinuing high-dose IV HBIG [20]. Marzano et al. reported that only 4 of 99 patients who received a combination of lamivudine and adefovir with high-dose intravenous HBIG for an indefinite period of time experienced HBV recurrence. Two of the 4 patients with recurrence in that study were noncompliant with NAT [6]. In addition, Gane et al. reported no HBV recurrence at a median of 11.7 months in 19 patients who received combination lamivudine and adefovir and only 7 days of IM HBIG [4]. Angus et al. reported the safe elimination of low dose, IM HBIG 12 months after LT with continuation of lamivudine and adefovir [5]. No patients had recurrent HBV DNA but one patient became HBsAg positive. There have been more recent studies reporting discontinuation of HBIG in selected patients, but the number of patients in these reports was limited. Noncompliance has been associated with HBV recurrence in several studies [16][21][22][23][24].

The optimal NAT regimen (monotherapy versus combination therapy) remains undefined. In our study, NAT with no HBIG or a short course of high-dose (10,000 IU) intravenous HBIG lasting between 1 week and 6 months was efficacious and safe. Only 4 of 28 patients (14%) had HBV recurrence a median 35 months after LT, and none of these were compliant with the prescribed treatment regimen. These 4 patients show that the risk of recurrence when patients are treated with combination NAT and minimal HBIG is still greater than zero [5][6][7][19]. Noncompliance with HBV prophylaxis seems to be the common factor associated with HBV recurrence across studies, and it was the only statistically significant predictor of HBV recurrence in our study (P < 0.0001). Although complete compliance to NAT utilization cannot be proven in patients without HBV recurrence, these patients had documented return clinical visits, NAT prescription renewals, and reported NAT utilization, unlike the patients with HBV recurrence, none of whom fulfilled any of these criteria.

Despite not reaching statistical significance, the 4 patients in our study with HBV recurrence had a higher median HBV DNA level at the time of LT (P > 0.20). With a larger sample size, these factors might have reached statistical significance. Recurrent HBV was successfully controlled in all 4 patients by re-initiating combination NAT. With regard to the secondary endpoints of the study, each of the HBIG groups had 1 graft loss unrelated to HBV recurrence, and 1 patient in Group 1 (no HBIG) died due to a cerebrovascular accident that was also unrelated to HBV recurrence. Previous studies have been limited in sample size and duration of the follow-up period. Meta-analyses and reviews of the literature on this topic have been limited to studies that have mainly utilized HBIG and lamivudine. Compared to earlier studies, the current study has the advantage of larger size, longer follow-up, shorter duration of HBIG administration, inclusion of treatment regimens that include no HBIG, and the inclusion of more than 1 type of NAT. Schiff et al. [19] included more patients who received no HBIG or only inpatient HBIG than our study did, but our study had a much longer follow-up time of 16.8 months for the no HBIG group (Group 1) and 23 months for the inpatient HBIG only group (Group 2). Our study also presents data on a larger number of patients [18] who received up to 6 months of monthly outpatient HBIG who were followed for a longer duration (a median of 42.9 months) as compared to the Schiff et al. paper.

Our study is limited, because it was a retrospective study of a heterogeneous group of patients from a single tertiary care center. In addition, the HBV prophylaxis regimen reflected a change in practice patterns over time, which was reviewed in a retrospective manner rather than determined in a prospective fashion. Finally, mutation testing was not routinely performed making those results unavailable. In conclusion, this study demonstrates that antiviral NAT with no or minimal HBIG is effective and safe in preventing HBV recurrence for up to 35 months after LT, provided that patients are compliant with antiviral therapy and follow-up care. The current study takes a definitive step forward in making the case for limiting HBIG to 1 week or eliminating its use when combination NAT is prescribed. Further study is needed, however, ideally in the context of a multi-center collaborative study.
